# Acceptance of COVID-19 Vaccine Booster Doses Using the Health Belief Model: A Cross-Sectional Study in Low-Middle- and High-Income Countries of the East Mediterranean Region

**DOI:** 10.3390/ijerph191912136

**Published:** 2022-09-25

**Authors:** Ramy Mohamed Ghazy, Marwa Shawky Abdou, Salah Awaidy, Malik Sallam, Iffat Elbarazi, Naglaa Youssef, Osman Abubakar Fiidow, Slimane Mehdad, Mohamed Fakhry Hussein, Mohammed Fathelrahman Adam, Fatimah Saed Alabd Abdullah, Wafa Kammoun Rebai, Etwal Bou Raad, Mai Hussein, Shehata F. Shehata, Ismail Ibrahim Ismail, Arslan Ahmed Salam, Dalia Samhouri

**Affiliations:** 1 Tropical Health Department, High Institute of Public Health, Alexandria University, Alexandria 21561, Egypt; 2 Department of Epidemiology, High Institute of Public Health, Alexandria University, Alexandria 21561, Egypt; 3 Health Affairs, Ministry of Health, Muscat 100, Oman; 4 Department of Pathology, Microbiology and Forensic Medicine, School of Medicine, The University of Jordan, Amman 11942, Jordan; 5 Department of Clinical Laboratories and Forensic Medicine, Jordan University Hospital, Amman 11942, Jordan; 6 Department of Translational Medicine, Faculty of Medicine, Lund University, 22184 Malmö, Sweden; 7 Institute of Public Health, College of Medicine and Health Sciences, United Arab Emirates University, AlAin 15551, United Arab Emirates; 8 Department of Medical-Surgical Nursing, College of Nursing, Princess Nourah bint Abdulrahman University, Riyadh 11671, Saudi Arabia; 9 School of Public Health and Research, Somali National University, Mogadishu P.O. Box 15, Somalia; 10 Physiology and Physiopathology Research Team, Research Centre of Human Pathology Genomics, Faculty of Sciences, Mohammed V University, Rabat BP 8007, Morocco; 11 Occupational Health and Industrial Medicine Department, High Institute of Public Health, Alexandria University, Alexandria 21526, Egypt; 12 Faculty of Pharmacy, University of Science and Technology, Khartoum P.O. Box 12810, Sudan; 13 Internal Medicine Department, Faculty of Medicine, Alexandria University, Alexandria 21526, Egypt; 14 Institute Pasteur de Tunis, Tunis 2092, Tunisia; 15 Department of Epidemiology and Population Health, American University of Beirut, Beirut P.O. Box 110236, Lebanon; 16 School of Pharmacy, Lebanese International University, Beirut P.O. Box 146404, Lebanon; 17 Clinical Research Administration, Alexandria Directorate of Health Affairs, Egyptian Ministry of Health and Population, Alexandria 21554, Egypt; 18 Harvard Medical School, Boston, MA 02115, USA; 19 Department of Family and Community Medicine, King Khalid University, Abha 62529, Saudi Arabia; 20 Biostatistics Department, High Institute of Public Health, Alexandria University, Alexandria 21561, Egypt; 21 Department of Neurology, Ibn Sina Hospital, Gamal Abdel Nasser Street, Sabah Medical Area, Safat 070001, Kuwait; 22 National Institute of Health, Islamabad 45320, Pakistan; 23 Emergency Preparedness and International Health Regulations, WHO EMRO (DS), P.O. Box 7608, Naser City 11371, Egypt

**Keywords:** COVID-19 vaccine, booster dose acceptance, health belief model, vaccine hesitancy, East Mediterranean region

## Abstract

Coronavirus disease (COVID-19) booster doses decrease infection transmission and disease severity. This study aimed to assess the acceptance of COVID-19 vaccine booster doses in low, middle, and high-income countries of the East Mediterranean Region (EMR) and its determinants using the health belief model (HBM). In addition, we aimed to identify the causes of booster dose rejection and the main source of information about vaccination. Using the snowball and convince sampling technique, a bilingual, self-administered, anonymous questionnaire was used to collect the data from 14 EMR countries through different social media platforms. Logistic regression analysis was used to estimate the key determinants that predict vaccination acceptance among respondents. Overall, 2327 participants responded to the questionnaire. In total, 1468 received compulsory doses of vaccination. Of them, 739 (50.3%) received booster doses and 387 (26.4%) were willing to get the COVID-19 vaccine booster doses. Vaccine booster dose acceptance rates in low, middle, and high-income countries were 73.4%, 67.9%, and 83.0%, respectively (*p* < 0.001). Participants who reported reliance on information about the COVID-19 vaccination from the Ministry of Health websites were more willing to accept booster doses (79.3% vs. 66.6%, *p* < 0.001). The leading causes behind booster dose rejection were the beliefs that booster doses have no benefit (48.35%) and have severe side effects (25.6%). Determinants of booster dose acceptance were age (odds ratio (OR) = 1.02, 95% confidence interval (CI): 1.01–1.03, *p* = 0.002), information provided by the Ministry of Health (OR = 3.40, 95% CI: 1.79–6.49, *p* = 0.015), perceived susceptibility to COVID-19 infection (OR = 1.88, 95% CI: 1.21–2.93, *p* = 0.005), perceived severity of COVID-19 (OR = 2.08, 95% CI: 137–3.16, *p* = 0.001), and perceived risk of side effects (OR = 0.25, 95% CI: 0.19–0.34, *p* < 0.001). Booster dose acceptance in EMR is relatively high. Interventions based on HBM may provide useful directions for policymakers to enhance the population’s acceptance of booster vaccination.

## 1. Introduction

Coronavirus disease 2019 (COVID-19), caused by severe acute respiratory syndrome (SARS-CoV-2), is still sweeping the globe. The pattern of infection and mortality due to COVID-19 differed significantly across countries [1,2], resulting in a high burden on healthcare facilities, the global economy, and social drawbacks [2,3]. As of 19 September 2022, there had been 611,421,786 confirmed cases of COVID-19 reported to the World Health Organization (WHO), including 6,512,438 deaths [4].

COVID-19 control is based on the strength of non-pharmaceutical interventions (NPIs) and the pace of infection development or decay, the speed with which the vaccine can be rolled out, vaccine targeting and uptake, and vaccine characteristics [5]. Although there have been international debates on the effectiveness of NPIs on viral transmission [6,7], many studies have proven the effectiveness of these measures on pandemic containment [8,9]. The cumulative effect of NPIs and immunization lowered the reproduction number (R_0_) by 53% (95% confidence interval: 42–62%), whereas NPIs and vaccination reduced transmission by 35% and 38%, respectively. So, both measures should be implemented simultaneously.

Globally, a total of 12,613,484,608 vaccine doses have been delivered as of September 12, 2022 [10], in an attempt to reduce infection through transmission and so as not to rely on herd immunity alone [11]. However, infection instances continued to be documented even after receiving two doses of the COVID-19 vaccine; this was referred to as “breakthrough infections,” and it has become a serious problem [12]. The current vaccine method may not establish a significant barrier against SARS-CoV-2 infections [13,14]. Research is still ongoing to determine the causes of breakthrough infections after vaccination. The substantial reduction in antibody titers over time following vaccination has been found to be one of the causes of such breakthrough [15]. Additionally, the multiple SARS-CoV-2 variants—including the most recent B.1.1.529 strain (in South Africa), the B.1.1.7 strain (in the United Kingdom), P.1 (in Brazil), and B.1.617 (in India)—increase the likelihood of breakthrough infections [13,16].

Recent clinical investigations have shown that the frequencies of confirmed COVID-19 and severe illness dramatically decreased with the third and fourth doses of inactivated or mRNA vaccine [17]. Other research also supported the booster vaccination’s ability to raise the titers of antibodies that neutralize SARS-CoV-2 variants significantly [18,19]. Consequently, experts in infectious diseases have carefully considered whether booster shots are necessary for all susceptible individuals or just a few vulnerable groups to enhance immunity levels and protect against new variations [20]. Hence, COVID-19 booster doses are expected to face rejection or hesitancy, which necessitates a thorough exploration of the underlying causes for such attitudes [21]. One of the ten threats to global health is vaccine hesitancy, which was widely prevalent in the context of COVID-19 vaccination [22,23,24,25]. Vaccine hesitancy is defined as the unwillingness or refusal to vaccinate despite the availability of vaccines [26]. This attitude represents a threat hindering the progress made in combating vaccine-preventable illnesses [27].

The East Mediterranean region (EMR) consists of 22 countries (i.e., Yemen, United Arab Emirates (UAE), Tunisia, Syrian Arab Republic, Sudan, Somalia, Qatar, Pakistan, Oman, Occupied Palestine Territory, Morocco, Libya, Lebanon, Kuwait, Jordan, Iraq, Iran, Egypt, Djibouti, Bahrain, and Afghanistan). These countries are of different income levels and health system capacities [28]. This gap is reflected in vaccination coverage; where the number of fully vaccinated subjects/100 population ranges from 1.58 in Yemen, 44.68, 54.0, and 59.4 in Jordan, Tunisia, and Pakistan respectively, peaking at 99.0 in the UAE. This vaccine coverage is much lower upon exploring the fraction of populations who received booster doses of COVID-19 vaccines, as it ranges from 0 to 51.6/100 people [10]. Regarding COVID-19 vaccine hesitancy in the EMR, an early study showed that only 26.7% of 4474 study participants from 13 countries were confident about the vaccination. The confidence was higher in high-income compared to low-and middle-income countries [29].

Studies about the acceptance of COVID-19 vaccine booster doses in the EMR are scarce. A study in Jordan reported that about 50% of the population have concerns about the side effects of vaccination, which might prevent them from receiving booster shots of vaccines, and 45.3% thought that receiving a third dose of the vaccine would exacerbate the side effects [30]. Higher acceptance of booster vaccination was reported among Saudi healthcare workers. Specifically, about 71.1% of 2059 healthcare workers accepted booster doses, with the following factors being significantly associated with vaccine booster dose acceptance: having comorbid conditions, higher educational level, high income, and being single [31].

In this study, we aimed to explore the acceptance of COVID-19 vaccine booster doses in low, middle, and high-income countries of the EMR and its determinants by using the Health Belief Model (HBM) constructions. The Health Belief Model is one of the most popular models for analyzing individuals’ behavior towards vaccination against COVID-19 [32]. The model hypothesizes that several variables influence the individual’s health-related behavior such as cues to action, self-efficacy, and perception of susceptibility, severity, benefits, and barriers [33]. In addition, we intended to identify the main causes of booster dose rejection and the sources from which the population received information about vaccination. The findings of this study can help in developing strategies for actions to upscale COVID-19 vaccine booster dose acceptance in EMRs.

## 2. Materials and Methods

### 2.1. Study Setting

A cross-sectional method was adopted to collect data from 14 EMR countries from March to June 2022. Based on the World Bank classification, the countries chosen were either low-income (Sudan, Somalia), middle-income (i.e., Egypt, Tunisia, Morocco, Libya, Jordan, Lebanon, and Pakistan), or high-income countries (Saudi Arabia, Kuwait, UAE, and Oman). An anonymous online survey was distributed by a team of researchers via social media platform and messaging platforms. 

### 2.2. Study Population and Sampling Methods

A convenience and snowball sampling techniques were used to recure the participants who must meet the following eligibility criteria for participation in this study: aged 18 years or older, had received two doses of the COVID-19 vaccine (or a single dose of the Johnson & Johnson vaccine), had a mobile phone or computer, educated to self-complete the survey, and residents in the EMR during the COVID-19 pandemic.

### 2.3. Sample Size

Epi-info 7 was used to estimate the minimum size of the required sample. With a 5% margin of error, 95% confidence level, 50% response rate, and a previously estimated rate of 60.0% for COVID-19 vaccine acceptance, a sample size of 642 (from the EMR) was considered sufficient. However, the sample size was doubled to compensate for stratification during the analysis of data and enhance the power of the study findings.

### 2.4. Tools of Data Collection

A questionnaire of two domains was created to collect the data. The first domain collected (i) sociodemographic and health condition data (i.e., age, sex, education, marital status, nationality, country of residency, body weight, height, previous COVID-19 infection, COVID-19 among relatives, history of chronic diseases, and immunocompromising diseases) and (ii) attitude towards the booster dose (I received the booster dose, planning to receive the booster dose, will not take any more doses).The second domain (HBM questionnaire) included 11 items that were used to assess the perceptions of COVID-19 infection and booster vaccination. 

The HBM consists of the following 6 domains: 

(i) Perceived susceptibility to COVID-19 infection: it consists of two items and refers to one’s beliefs about the chances of worsening his/her health condition. (ii) Perceived severity of COVID-19 which refers to one’s feeling about the seriousness of his/her health condition, ability to worsen, or failure to treat the illness. This item also consists of two items. Moreover, the perceived severity domain includes one’s evaluation of medical and clinical outcomes such as fatality, infirmity, and discomfort, as well as potential consequences on her/his daily social activities such as the effects of their medical condition on work productivity, family activities, and social interactions and relations. According to the HBM, perceived threats were defined as the combination of perceived susceptibility and perceived severity. Thus, the first and second domains can be applied under one domain labeled “perceived threats’’. (iii) Perceived benefit of COVID-19 booster indicates that the individual’s beliefs about the perceived benefits of the various available actions for reducing the disease threat are influenced by his/her perception of the threat, regardless of whether or not that perception ended with a behavior change. This domain consists of three items, (iv) a perceived barrier to receiving COVID-19 booster doses, which indicates the impending negative consequences and aspects of a particular health action that may considerably act as obstacles to engagement in recommended health behaviors. This domain consists of three items. As per the HBM, perceived susceptibility and perceived benefits could only be potentiated by other independent factors, particularly by clues used to prompt actions, namely “cues to action”, such as physical condition, or by environmental factors, such as media. (v) self-efficacy has been elaborated on by the HBM as an essential factor that may encourage certain behavior. Moreover, it is defined as “the conviction that one can successfully execute the behavior required to produce the outcomes” [34]. 

The responses to the questionnaire’s items were aggregated as follows: high (high/very high), neutral, and low (low/very low). All domains of the HBM questionnaire had an acceptable level of validity as Cronbach’s Alpha value was ≥0.6, except the perceived barrier was 0.59 [35]. Of the six dimensions that make up the HBM model, five of which are positive and one of which is negative. According to the model, behavior is more likely to be present among people who may think there will be little or no obstacles to adopting behaviors; that it will lower the chance of a negative health outcome; that it will have a significant negative impact on their health; and if they think they are vulnerable to it [36].

The questionnaire was administered in Arabic (for the general population) and English (for healthcare professionals or others) to eliminate the difficulties of language as previously suggested [35]. The face validity of the questionnaire was investigated before administration.

### 2.5. Plan of Data Collection

**Figure 1:**
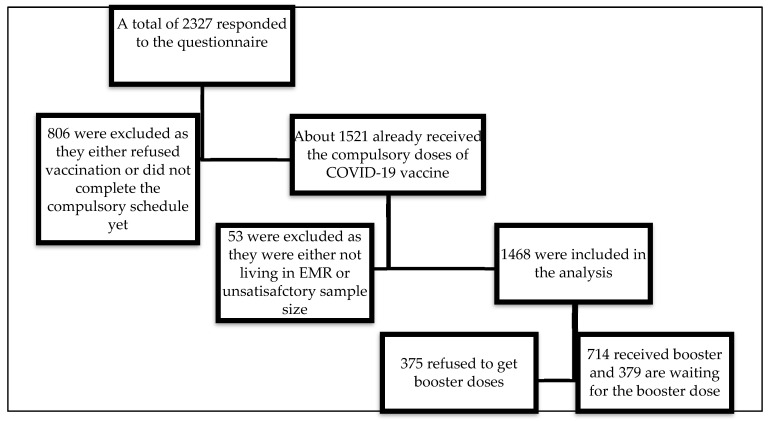
The flow chart of the sampling process.

An electronic form of the questionnaire was designed using Google Forms and distributed via different social media (Facebook and Twitter) and messaging platforms (WhatsApp and emails) from March to June 2022. Before actual data collection, the research team tested the feasibility and accessibility of the online tool in a pilot study. Each collaborator was asked to submit at least two responses to determine the time required for completing the survey and the feasibility of the study. A total of 2327 individuals responded to the questionnaire. About 806 (34.6%) were excluded as they either did not receive the vaccination at all or did not complete the compulsory schedule of COVID-19 vaccination. Another 53 (2.3%) responses were omitted as the responders were not living in EMR countries or the responses were unsatisfactory. Finally, 1468 were included in the analysis; 375 (25.5%) refused to get the booster, 739 (50.3%) received booster doses, and 387 (26.4%) were willing to get the booster doses. In Figure 1, we aggregated data on those who have already received booster doses and those who are willing to receive booster doses, who represented 74.5% (1093/1468).

### 2.6. Ethical Considerations and Approval

The Ethics Committee of the High Institute of Public Health, Alexandria University, Egypt approved the study. Information about the study’s purpose, anonymity, confidentiality, voluntary participation, and privacy statements was provided on the survey cover page to all participants who were prompted “to agree” or “not to agree” to participate in the study. Those who clicked “I agree to participate” were able to access the questionnaire. Data were protected and saved in a password-accessible computer available only to the principal investigator.

### 2.7. Statistical Analysis

The data were managed and analyzed using the R 4.2.1 software (R Foundation for Statistical Computing, Vienna, Austria). Numerical variables were presented using mean ± standard deviation (SD), whereas nominal and categorical variables were expressed as a percentage (%). A Chi-Square test was used to assess the association between the nonnumerical variables, and the responses were categorized into Yes or No based on receiving COVID-19 booster doses. An independent t-test was performed to compare the difference between the means of two independent groups. Cronbach’s alpha test was deployed to assess the internal consistency of the domains and their items. A binary logistic regression analysis was conducted to estimate the significant predictors’ odds ratios, and a confidence interval of 95% (OR, 95% CI) was reported. The dependent variable was the actual or virtual acceptance of COVID-19 booster vaccination, which was defined based on the following questions: “Have you received the booster dose of COVID-19 vaccine” or “Will you get the booster dose of COVID-19 vaccine” (Yes/No)”. A *p*-value < 0.05 was considered statistically significant.

## 3. Results

### 3.1. Respondents’ Sociodemographic Characteristics

The mean age of the respondents was 36.53 ± 13.45, ranging from 18–88 years; females represented 62.7% (920); more than half of them were married 55.2%, (811); nearly one half 48.0% (705) had a university degree; about one-third 35.8% (526) were working in the medical field; 16.1% (237) had chronic diseases; and those who had COVID-19 infection before accounted for 51.1% (750); those who had relatives that had COVID-19 infection represented 86.4% (1268); and those with relatives who were immunocompromised and living in the vicinity of the participant made up 14.3% (210) of respondents (Table 1).

### 3.2. COVID-19 Booster Dose Acceptance in Low-, Middle, and High-Income Countries

**Figure 2:**
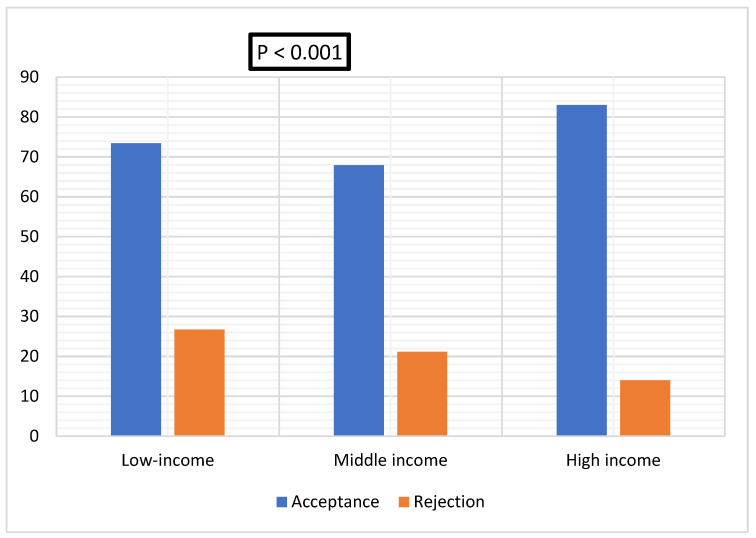
Acceptance of COVID-19 vaccine booster dose across different countries of the East Mediterranean Region based on their income level.

There was a significant difference in booster dose acceptance among the included EMR countries, being the highest in low-income countries at 73.4% (141/192), and lowest in middle-income countries at 17.0% (480/707) at *p* < 0.001 Figure 2.

### 3.3. Characteristics of Vaccinated and Non-Vaccinated Participants

Acceptance of the COVID-19 vaccine was reported more frequently among males than females (79.4% vs. 71.5%), *p* = 0.001. The mean age of respondents who accepted vaccination was significantly older than those who accepted vaccination (37.5 ± 13.8 vs. 33.9 ± 12.0, respectively, *p* < 0.001). The mean BMI of respondents who accepted booster doses tended to be higher than those who rejected (26.2 ± 5.5 vs. 25.6 ± 5.8). However, this difference was insignificant (*p* = 0.075). Being married was significantly associated with booster dose acceptance, as 77.7% of those who were married versus 69.6% of those who were single accepted booster dose, with *p* = 0.003. About 71.1% (533/750) of respondents -19 previously infected with COVID vs. 78.0 (560/718) of non-previously infected respondents accepted booster doses (*p* = 0.003). About 75.5% (959/1258) of those who accepted vaccination had no immunocompromised person living in the same context compared to 68.1% (143/210) of participants who had immunocompromised relatives (*p* = 0.028). Neither occupation, chronic diseases, nor having a relative who was infected with COVID-19 were associated with booster dose acceptance (Table 2).

### 3.4. Leading Causes behind Booster Dose Rejection

**Figure 3:**
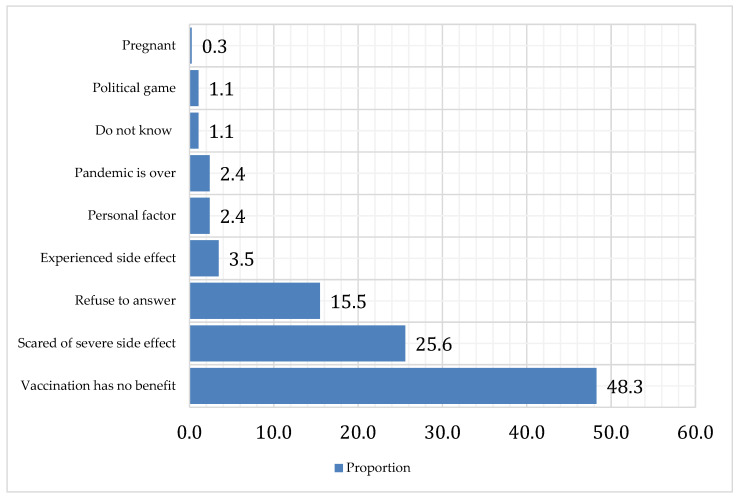
The leading causes behind COVID-19 booster dose vaccine rejection among the participants.

The leading causes behind booster dose rejection were the beliefs that the booster vaccine has no benefit 48.35% (181/375) and that it has severe side effects 25.6% (96/375). About 15.5% (58/375) refused to answer this question. Figure 3.

### 3.5. Source of Information about COVID-19

Asking participants about the primary sources of information about COVID-19 disease and vaccination revealed that the most frequently used sources were social media 56.0% (646/1468), the Center for Diseases Prevention and Control (CDC) website 53.6% (787/1468), followed by the Ministry of Health (MOH) website 41.3% (905/1468). However, 79.3% (718/905) of those who did follow the information provided by the MOH website accepted booster doses compared to 66.6% (375/863) of those who did not follow this source of information; this difference was statistically significant (*p* < 0.001) (Table 3).

### 3.6. Determinants of Booster Dose Acceptance

All domains of the HBM (i.e., perceived susceptibility, perceived severity, perceived benefit, perceived barriers, and perceived efficacy) were statistically significant across vaccinated and unvaccinated groups (*p* < 0.05) except for question 13 (cues to action) as shown in Table 4. The HBM questionnaire has a Cronbach’s alpha of 0.68, and it ranged from 0.63 to 0.73 for each question.

#### Determinants of Booster Dose Acceptance Using Multivariable Regression Analysis

**Figure 4:**
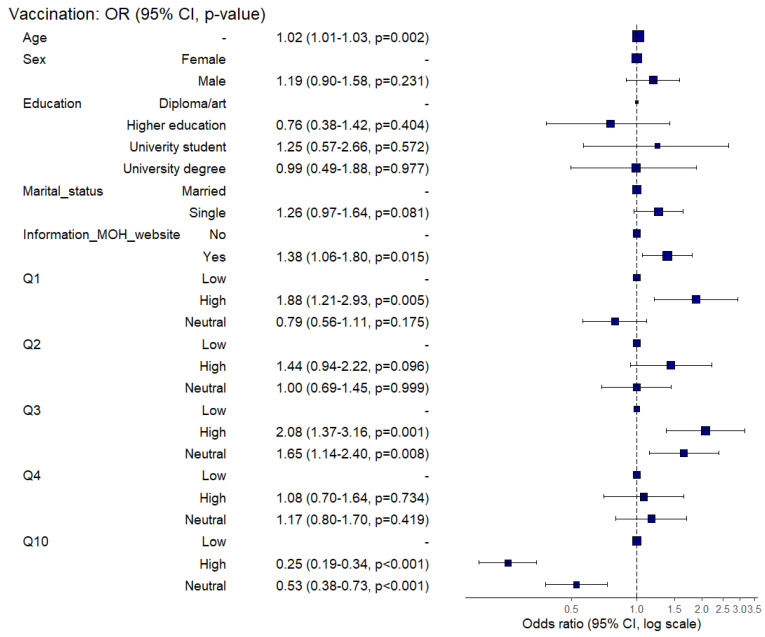
Forest plot of the adjusted odds ratio s; the dependent factor is vaccine acceptance.

Figure 4 depicts a multivariable regression analysis which revealed that age (OR = 1.02, 95% CI: 1.01–1.04, *p* = 0.002), information source as Ministry of Health and population (OR = 1.38, 95% CI: 1.6–1.80, *p* = 0.015) were significant determinants of COVID-19 booster dose acceptance. Regarding the HBM, perceived susceptibility to COVID-19 infection (OR = 1.88, 95% CI: 1.21–2.93, *p* = 0.005), perceived severity of COVID-19 (OR = 2.08, 95% CI: 137–3.16, *p* = 0.001), and perceived risk of side effects (OR = 0.25, 95% CI: 0.19–0.34, *p* < 0.001) were the significant predictors of COVID-19 booster dose acceptance.

## 4. Discussion

### 4.1. Booster Dose Acceptance

The present study demonstrated a high COVID-19 vaccination booster acceptance rate (74.46%) among the population in the EMR. This figure is substantially higher than a recently published study by Abuzaid et al., [37]. They reported that 60.2% of the respondents accepted the booster dose of the COVID-19 vaccine, and 20.4% were hesitant to receive the vaccination. We hypothesize that this difference may be related to the various research settings and scenarios. They only conducted their study in five countries (Egypt, Iraq, Palestine, Saudi Arabia, and Sudan) in November and December 2021. In comparison, we included around 14 countries from different time periods. Furthermore, our sample was more representative as we included countries from North Africa (Tunisia and Morocco). Similarly, a higher booster dose acceptance rate (84.4%) was observed among the Chinese population aged 18–59 years [38]. Interestingly, Qin et al., [39] found that 93.7% of study participants accepted the booster dose of the vaccination. It is important to note that the greater public expectations for boosters to meet the new challenges posed by variant strains and the observed rising trend in COVID-19 vaccine acceptance may contribute to the higher booster acceptance rate. On other hand, Al-Qerem et al., [40] reported that almost half of the participants, 44.6% (n = 915), of Jordanian adults who have completed their current immunization schedule expected to get the COVID-19 booster dosage.

### 4.2. Acceptance Rate in Low-Middle, and High-Income Countries

In the current study, vaccine acceptance was significantly higher in high-income countries compared to low- and middle-income countries. Indeed, high-income countries received more doses from manufacturers in order to vaccinate a larger proportion of their population. On the other hand, Arce et al., [41] reported that participants in low- and medium-income countries have a stronger readiness to take vaccines but limited access to them, whereas high-income countries have adequate access to vaccines but high levels of hesitation against taking a vaccination. We speculate that in this work we already assessed the actual booster and intentional acceptance of booster doses, while Arce and his colleagues assessed the intentional acceptance of the vaccine. Moreover, the context was different as we assessed booster dose acceptance while they measured acceptance to first and second doses.

### 4.3. Determinants of Booster Dose Acceptance

In our univariate analysis, being female, younger age, being single, having a university degree or being postgraduate, and previous COVID-19 infection were significantly associated with booster dose acceptance. However, in multiple regression, age and the information provided by the Ministry of Health were the only significant determinants of vaccine acceptance. On the other hand, Lai et al., [38] reported that increased odds of booster acceptance were associated with prior COVID-19 vaccination, being young (18–30 vs. 41–50), lower educational attainment, employment, and belonging to a priority group for vaccination. The differences in sociodemographic traits between vaccination acceptability and booster acceptability require more research. This study’s findings on the relationships between sociodemographic traits and booster acceptance could be a starting point for developing booster-targeted immunization programs to increase coverage.

### 4.4. Health Belief Model

We used the HBM to predict booster dose vaccine acceptance in the current study, and the questionnaire has a satisfactory level of internal consistency among the studied sample. In line of previous studies, our study findings showed that the HBM aspects served as a valuable framework for this study’s assessment of participants’ attitudes concerning a booster dose of the COVID-19 vaccination. In the univariate analysis, except for question 13, all items of all constructs of the HBM were significantly associated with booster dose acceptance. In contrast, in the multivariable analysis, perceived susceptibility, perceived severity, and perceived barriers were significant predictors of booster dose acceptance. For example, Wang et al., [42] found that five dimensions of the HBM were significantly associated with COVID-19 vaccine acceptance in China. Additionally, a study conducted by Lai et al. [38] suggested that perceived benefits and perceived barriers to vaccination were important dimensions associated with accepting COVID-19 boosters. On the contrary, Quin et al., [39] found that cues to action were the most significant predictor of vaccine acceptance. So, we can conclude that the HBM can be used to assess the population’s beliefs towards the acceptance of COVID-19 booster doses.

### 4.5. Causes of COVID-19 Vaccine Rejection

Our study aimed to look into the individuals who were hesitant to receive a COVID-19 vaccine booster to better understand the contributing factors. Interestingly, we found that most of them thought that booster doses had no benefit, and about one-fourth refused vaccination as they witnessed severe side effects among vaccinated individuals. A prior study by Al-Qerem et al., [40], similarly, found that the most frequently stated reasons for participants’ reluctance to receive the COVID-19 vaccine booster dose were that they believed the benefits of the booster dose were not scientifically proven, followed by the belief that there will be no need to take the booster dose for at least a year as they had received it recently, and there is no need for a booster as they were infected with COVID-19. 

In the same vein, Lin et al., [43] found that among the Chinese population, those who were hesitant to receive booster doses of the vaccination expressed concern about the vaccine’s side effects. However, data from clinical trials proved vaccine efficacy and safety [44,45,46].

### 4.6. Source of Information about Vaccination

Infodemic have a negative impact on the COVID-19 pandemic in terms of the increased frequency of self-treatment, [47] non-compliance to NPIs, and vaccine rejection [48]. It is worth noting that, in this study, social media was the most important source of information about COVID-19 and vaccination, while the information provided by the Ministry of Health had a significant effect on vaccine acceptance in the multivariable analysis. These commonly used media channels can be utilized to deliver accurate health messages about the safety and immunogenicity of vaccination. This approach may reduce the fear of those who were hesitant. Moreover, these media can reflect the attitude of the population towards immunization [49]. One of the important messages that should be delivered is that the principal objective of vaccination during the COVID-19 pandemic continues to prevent hospitalizations, life-threatening illnesses, and death. Therefore, a third dose of the COVID-19 vaccination may only be required if evidence of insufficient protection against these negative outcomes over time is present. A third dosage may be necessary depending on the target group, the type of vaccine used, the prevalence of SARS-CoV-2, particularly important SARS-CoV-2 variants, and the level of exposure [50]. Based on this fact, Jairoun et al., [51] assessed the population’s knowledge and attitude, and the acceptance of the COVID-19 vaccination booster in the UAE. The average knowledge score and attitude score among 642 participants were 44.6% and 70.2%. Knowledge level was higher among postgraduates, healthcare workers, participants with relatives infected with COVID-19, participants infected with COVID-19, and participants who had received the first two doses of the COVID-19 vaccine [51].

Finally, although the COVID-19 pandemic has been successfully controlled in many countries, SARS-CoV-2 still seriously threatens the world’s health [52]. Indeed, vaccine acceptance has a detrimental effect on preventing and controlling infectious diseases and makes it harder to build herd immunity. Thus, it is essential to lower people’s vaccine hesitancy to develop an immunological defense against SARS-CoV2 infections [53]. Understanding people’s vaccination intentions is a crucial step to increasing immunization rates globally, especially in poorer nations [52].

### 4.7. Strengths and Limitations

There are several limitations to this study. First, due to the intrinsic disadvantages of cross-sectional online surveys, a sampling bias may exist to limit the representativeness of the results [54]. Second, self-reported data could be skewed by recall bias and a propensity to present socially desirable outcomes. Third, study participants were recruited through a non-random sampling method; this may affect the external validity and generalization. Fourth, the sample collected from countries may be not representative of the situation of the country; however, the objective was to assess whether there was a difference among countries based on their socioeconomic level or not. Fifth, about one-third of respondents were working in the medical field. This may represent a source of selection bias. Finally, there exist inherent problems with the HBM itself. The HBM does not take into consideration a person’s attitudes, beliefs, or other personal factors that influence whether they accept a healthy behavior. Habitual behavior is not taken into account by the HBM, which may influence the decision to adopt a suggested action (e.g., smoking). The HBM does not account for actions taken for social acceptance, environmental, or economic considerations that can support or discourage the advised course of action. Furthermore, the HBM assumes everyone has equal access to knowledge about the condition or disease. However, to our knowledge, this is the first study in the EMR that used the HBM to assess vaccine acceptance in the region. This study included 14 of the 22 countries in the region based on different income levels reflecting the heterogeneity of the population in the region.

## 5. Conclusions

A third dose of the COVID-19 vaccination was acceptable to 74.47% of the EMR inhabitants in this regional cross-sectional study. Acceptance behaviors, increased age and the HBM construct were highly associated. However, there may be some reluctance due to public skepticism regarding the safety and efficacy of current vaccines in practical settings. Our findings can therefore assist policymakers in creating more precise and scientific roll-out plans for the third COVID-19 vaccine, which is crucial when a further outbreak is still conceivable.

## Figures and Tables

**Table 1:** Sociodemographic criteria and medical conditions, n = 1468.

Variables		n (%)
Sex	Males	548 (37.3)
	Females	920 (62.7)
Age	Mean ± SD (min-max)	36.53 ± 13.45 (18.0–88.0)
Marital status	Single	596 (40.6)
	Married	811 (55.2)
	Divorced	42 (2.9)
	Widow	19 (1.3)
Education	Diploma	87 (5.9)
	Secondary education	171 (11.6)
	University students	705 (48.0)
	Postgraduate	505 (34.4)
Working	I do not work	186 (12.7)
	Retired	50 (3.4)
	Students	293 (20.0)
	Working in the medical field	526 (35.8)
	Working outside the medical field	413 (28.1)
Chronic Disease	Yes	237 (16.1)
	No	1231 (83.9)
Previous COVID-19 infection	Yes	750 (51.1)
	No	718 (48.9)
A relative had a COVID-19 infection	Yes	1268 (86.4)
	No	200 (13.6)
Immunocompromised relative	Yes	210 (14.3)
	No	1258 (85.7)

**Table 2:** Comparison of different characteristics among vaccinated and non-vaccinated participants.

Dependent (Vaccination)		Total	Accept Vaccination (n = 1093)		Reject Vaccination (n = 375)		*p*
			n	%	n	%	
Sex	Female	920	658	71.5	262	28.5	0.001
	Male	548	435	79.4	113	20.6	
Age	Mean ± SD		37.5 ± 13.8		33.9 ± 12.0		<0.001
Body mass index	Mean ± SD		26.2 ± 5.5		25.6 ± 5.8		0.075
Marital status	Married	811	630	77.7	181	22.3	0.002
	Single ^#^	657	463	69.6	194	30.4	
Education	Secondary education	171	143	83.6	28	16.4	0.002
	Diploma/Art	87	73	83.9	14	16.1	
	University	705	517	73.3	188	26.7	
	Higher Education	505	360	71.3	145	28.7	
Previous COVID-19 infection	No	718	560	78.0	158	22.0	0.003
	Yes	750	533	71.1	217	28.9	
Immunocompromised relative	No	1258	950	75.5	308	24.5	0.028
	Yes	210	143	68.1	67	31.9	

^#^ Single includes widow, divorced, and single.

**Table 3:** Source of information about COVID-19.

Dependent: Vaccination		Total (n = 1468)	Accept Vaccination		Reject Vaccination		*p*
			n	%	n	%	
Social media	No	646 (44.0)	469	72.6	177	27.4	0.166
	Yes	822 (56.0)	624	75.9	198	24.1	
Relative and friends	No	1122 (76.4)	835	74.4	287	25.6	1
	Yes	346 (23.6)	258	74.6	88	25.4	
Literature	No	816 (55.6)	613	75.1	203	24.9	0.551
	Yes	652 (45.4)	480	73.6	172	26.4	
Ministry of Health website	No	863 (58.7)	375	66.6	188	33.4	<0.001
	Yes	905 (41.3)	718	79.3	187	20.7	
CDC website	No	681 (46.4)	854	74.0	300	26.0	0.492
	Yes	787 (53.6)	239	76.1	75	23.9	
WHO website	No	1154 (78.6)	492	72.3	189	27.8	0.081
	Yes	314 (21.4)	601	76.34	186	23.6	
Other	No	1452 (98.9)	1082	74.6	369	25.4	0.517
	Yes	17 (1.1)	11	64.7	6	35.3	

CDC: Center for Diseases Prevention and Control, the *p*-value was significant if <0.05, and the chi-square was the used statistical test.

**Table 4:** Perception of the booster doses based on the health belief model.

Dependent: Vaccination	Question/Category		Total	Accept Vaccination		Reject Vaccination		*p*	Cronbach Alpha
			n	n	%	n	%		0.68
Perceived susceptibility	Q1: I think there is a risk of COVID-19 infection	High	601	74	12.3	527	87.7	<0.001	0.65
		Low	387	137	35.4	250	64.6		
		Neutral	480	164	34.2	316	65.8		
	Q2: I think COVID-19 variants have a higher risk of infection than the existing strains	High	600	93	15.5	507	84.5		0.65
		Low	447	157	35.1	290	64.9		
		Neutral	421	125	29.7	296	70.3		
Perceived severity	Q3: I think COVID-19 infection is a severe disease	High	709	113	15.9	596	84.1	<0.001	0.65
		Low	304	127	41.8	177	58.2		
		Neutral	455	135	29.7	320	70.3		
	Q4: I agree that COVID-19 variants can cause more severe illness than the existing strains	High	553	112	20.3	441	79.7	<0.001	0.66
		Low	391	138	35.3	253	64.7		
		Neutral	524	125	23.9	399	76.1		
Perceived benefit	Q5: I believe the COVID-19 boosters are effective against early circulating COVID-19 strains	High	654	43	6.6	611	93.4	<0.001	0.63
		Low	399	219	54.9	180	45.1		
		Neutral	415	113	27.2	302	72.8		
	Q6: I believe the COVID-19 boosters are effective to extend protection against COVID-19 infection.	High	699	54	7.7	645	92.3	<0.001	0.63
		Low	369	221	59.9	148	40.1		
		Neutral	400	100	25.0	300	75.0		
	Q7: I believe the COVID-19 boosters are effective against COVID-19 variants	High	612	39	6.4	573	93.6	<0.001	0.63
		Low	421	229	54.4	192	45.6		
		Neutral	435	107	24.6	328	75.4		
Perceived barriers	Q8: I think COVID-19 vaccine boosters are safe	High	594	39	6.6	555	93.4	<0.001	0.65
		Low	426	228	53.5	198	46.5		
		Neutral	448	108	24.1	340	75.9		
	Q9: I am worried about the serious adverse reaction after vaccination	High	411	197	47.9	214	52.1	<0.001	0.67
		Low	711	98	13.8	613	86.2		
		Neutral	346	80	23.1	266	76.9		
	Q10: I know persons had severe side effects after being vaccinated	High	362	158	43.6	204	56.4	<0.001	0.68
		Low	788	126	16.0	662	84.0		
		Neutral	318	91	28.6	227	71.4		
Perceived Efficacy	Q11: It is easy for me to get the COVID-19 vaccine if I wanted to	High	1056	251	23.8	805	76.2	0.042	0.67
		Low	120	35	29.2	85	70.8		
		Neutral	292	89	30.5	203	69.5		
Cues to action	Q12: Did you use to have confirmed or suspected cases in your daily close contacts?	No	772	595	77.1	177	22.9	0.018	0.70
		Yes	696	498	71.6	198	28.4		
	Q13: Do you know about the following COVID-19 variants?	All Types	321	249	77.6	72	22.4	0.257	0.73
		Four Types	115	82	71.3	33	28.7		
		I Don’t Know	160	123	76.9	37	23.1		
		One Type	354	270	76.3	84	23.7		
		Three Types	198	144	72.7	54	27.3		
		Two Types	320	225	70.3	95	29.7		

*p*-value was significant if <0.05, and chi-square was the used statistical test.

## Data Availability

Data are available upon request by mailing the first author.
